# Premature mortality patterns among American Indians in North Dakota, 2010–2019 (pre-pandemic)

**DOI:** 10.3389/fpubh.2025.1395399

**Published:** 2025-02-26

**Authors:** Melanie Nadeau, Joel S. Steele, Amber Lyon-Colbert, Allison Kelliher, Joshua Barnett, Andria Begay, Donald Warne

**Affiliations:** ^1^School of Medicine and Health Sciences, University of North Dakota, Grand Forks, ND, United States; ^2^Center for Indigenous Health, Johns Hopkins University, Baltimore, MD, United States

**Keywords:** premature mortality, American Indians, mortality patterns, North Dakota, health disparities

## Abstract

**Background:**

According to the National Center for Health Data, in 2017 American Indians in North Dakota experience the highest age-adjusted mortality rate in the United States. Data shows that the age-adjusted death rate for all North Dakotans has steadily declined since 1979. However, mortality remains high among American Indians in North Dakota.

**Purpose:**

Assess pre-pandemic disparities and age-specific mortality patterns to better inform and guide communities and providers on prevention, planning, and policy efforts to advance health equity.

**Methods:**

Death certificate data from 2010–2019 were obtained and analyzed, evaluating the decade prior to the COVID-19 pandemic in order to assess pre-pandemic age-specific mortality rates and rate ratios for 3,369 American Indian and 57,778 White residents in North Dakota. Premature mortality is defined as death <65 years of age.

**Results:**

The median age at death for American Indian males in North Dakota from 2010 to 2019 was 55 compared to 77 years for White males, and the median age at death for American Indian females was 62 compared to 85 years for White females. Consistent patterns for leading causes of death showed that American Indians suffer disproportionately compared to White residents in North Dakota. The three leading causes of death for American Indians in North Dakota, accounting for approximately 55.4% of all deaths were diseases of the heart (21.7%), cancer (18.5%), and accidents (15.2%). From 2010 to 2019, all cause mortality rates were higher in every age and sex stratum for American Indians compared to White residents in North Dakota. American Indians die 22.5 years younger on average compared to White residents in North Dakota starting at birth and continuing over the lifespan.

**Conclusion:**

To best address the health and wellbeing of the American Indian population in North Dakota, multisectoral efforts focused on prevention, improved policies, and cultural humility and safety in the health systems are needed. Solutions should center American Indian voices, cultures and spaces, and include Tribes, Tribal organizations, Tribal health, Indian Health Service and Urban Indian Health Centers. Additionally, standards for death certificates in the United States could benefit populations by accurately reflecting race and rates of illness and death.

## 1 Introduction

Mortality rates for American Indians and Alaska Natives vary greatly across the United States (U.S.). The differences between American Indian populations and other racial or ethnic groups also varies by state. For example, data shows age-adjusted mortality rates for American Indians in Illinois, New Jersey, and Texas are far lower than other groups in those states. However, the same data shows mortality rates for American Indians are far higher than other groups in states such as North Dakota, South Dakota, and Montana (1).

There are four federally recognized Tribes that are headquartered in North Dakota with American Indians representing approximately 5% of the population throughout the state (2). This includes the Three Affiliated Tribes (Mandan, Hidatsa, and Arikara Nation), Spirit Lake Nation, Standing Rock Sioux Tribe, and Turtle Mountain Band of Chippewa Indians. Health disparities impacting the health and well-being of the American Indian population in North Dakota include, but are not limited to, diabetes, cancer, heart disease and unintentional injuries (3).

According to the 2021 North Dakota State Health Assessment, the ten leading causes of death for American Indians in North Dakota from 2017 to 2020 were diseases of the heart, cancer, accidents, cirrhosis, diabetes, COVID-19, chronic obstructive pulmonary disease, cerebrovascular, suicide, and septicemia (3). Premature deaths in North Dakota (deaths prior to age 65) accounted for 57% of American Indian deaths compared to 21% for the state population (3). From 2017 to 2020, the median age of death for American Indians’ in North Dakota was 57 (males and females combined), which is 20 years younger, on average, than other races in the state (3). Data show that the age-adjusted death rate for all North Dakotans has steadily declined since 1979, however mortality remains high among American Indians (1).

The purpose of this study is to assess mortality disparities for American Indians living in North Dakota the decade prior to the COVID-19 pandemic (2010–2019).

## 2 Methods

Publicly available population mortality data from 2010 to 2019 were obtained from the North Dakota Department of Health & Human Services Division of Vital Records (4). Data from the decade prior to the COVID-19 pandemic was analyzed in order to assess pre-pandemic disparities. During those 10 years, 3,369 American Indian and 57,778 White residents died (4). Upon death, funeral home directors collected demographic information and medical providers completed the death certificate, recording the date, time, and cause of death. The causes of death were coded by the Division of Vital Records using ICD-10 (4). Annual denominator data for mortality rate calculations came from the United States Census Bureau, Decennial Census (5). Classification of American Indians included “race alone or race in combination” with one or more other races (5). If American Indian was reported on the death certificate, the individual was classified as American Indian for this study, regardless of if other races were reported. All analysis were conducted using R version 4.4.1 and epitools version 0.5-10.1 package to compute rates and rate ratios (RRs) between American Indian and White residents in North Dakota from 2010 to 2019. No ethical approval was needed to conduct this study.

## 3 Results

**Figure 1:**
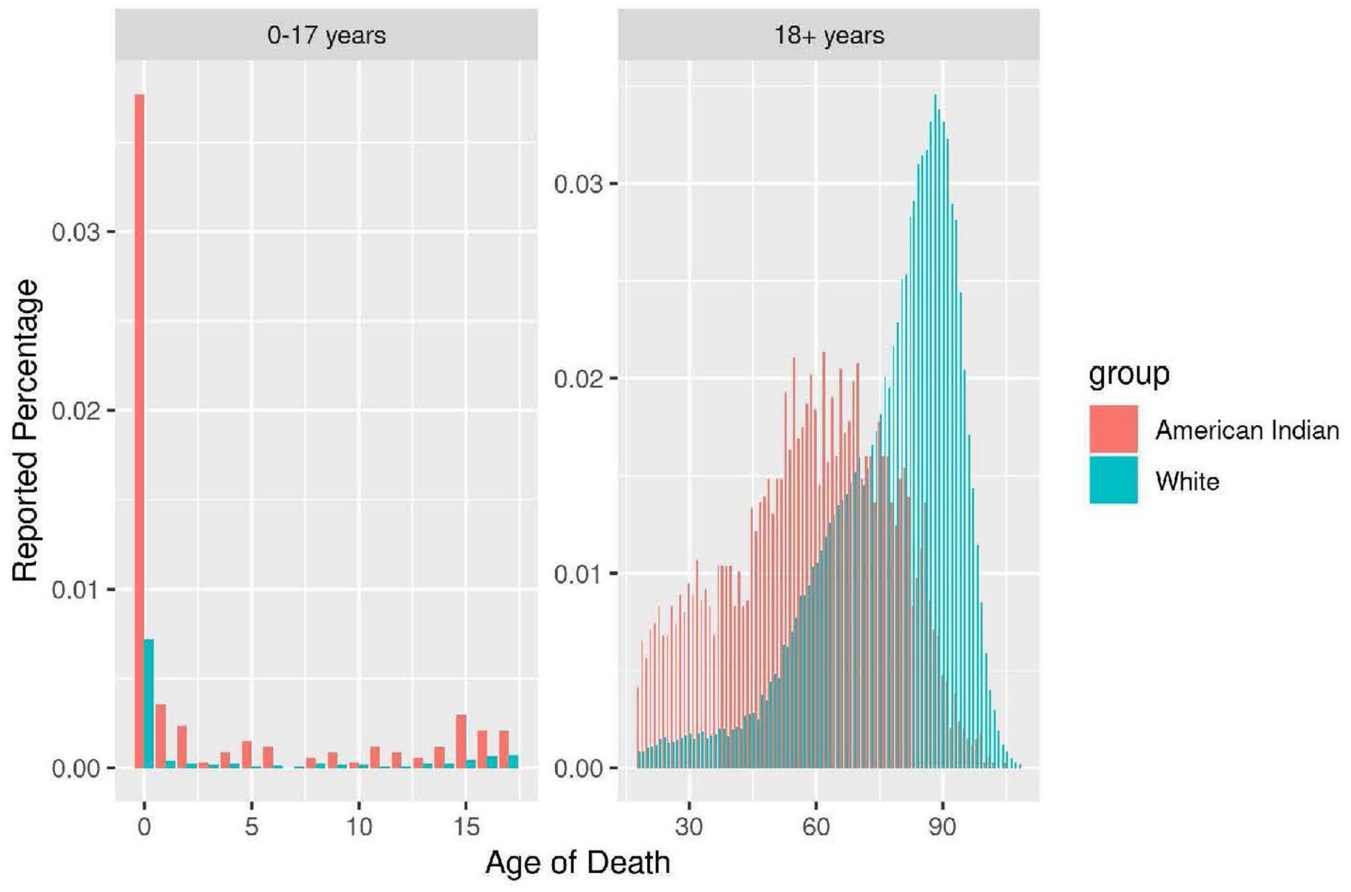
Age-specific percent of reported death 0–17, 18+ years, North Dakota 2010–2019.

**Figure 2:**
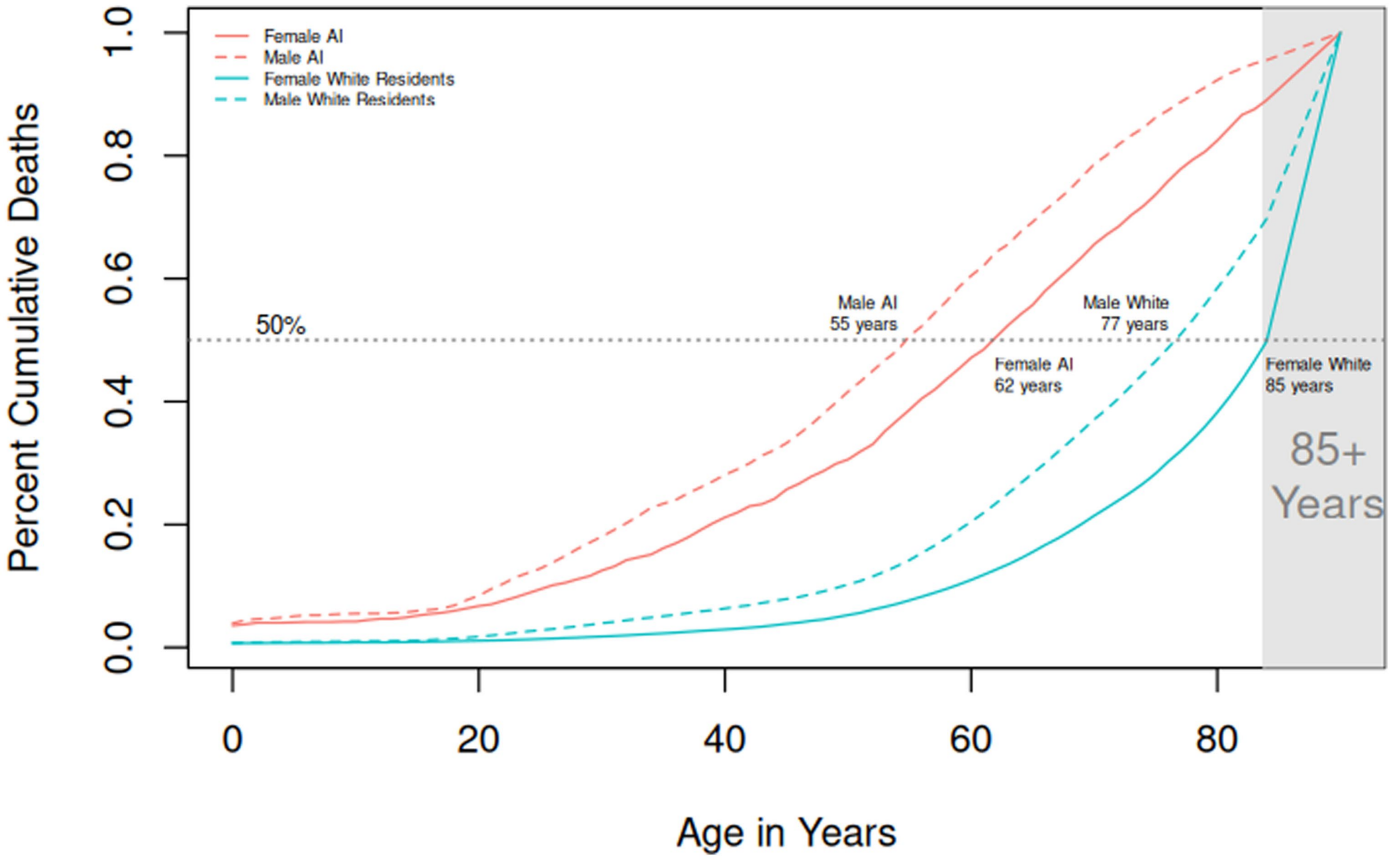
Percent cumulative death by age, North Dakota 2010–2019. AI represents American Indian.

Age-specific percent of reported death for American Indian versus White residents ages 0–17 and 18+ years in North Dakota from 2010 to 2019 is shown in Figure 1. This figure demonstrates that American Indians in North Dakota are more likely to die at a younger age than White residents. Percent cumulative death by age for American Indian versus White residents in North Dakota from 2010 to 2019 is shown in Figure 2. The median age of death in North Dakota for American Indian males is 55 years compared to 77 years for White males. The median age of death in North Dakota for American Indian females is 62 years compared to 85 years for White females. American Indians die an average of 22.5 years younger compared to White residents starting at birth and continuing over the lifespan.

**Figure 3:**
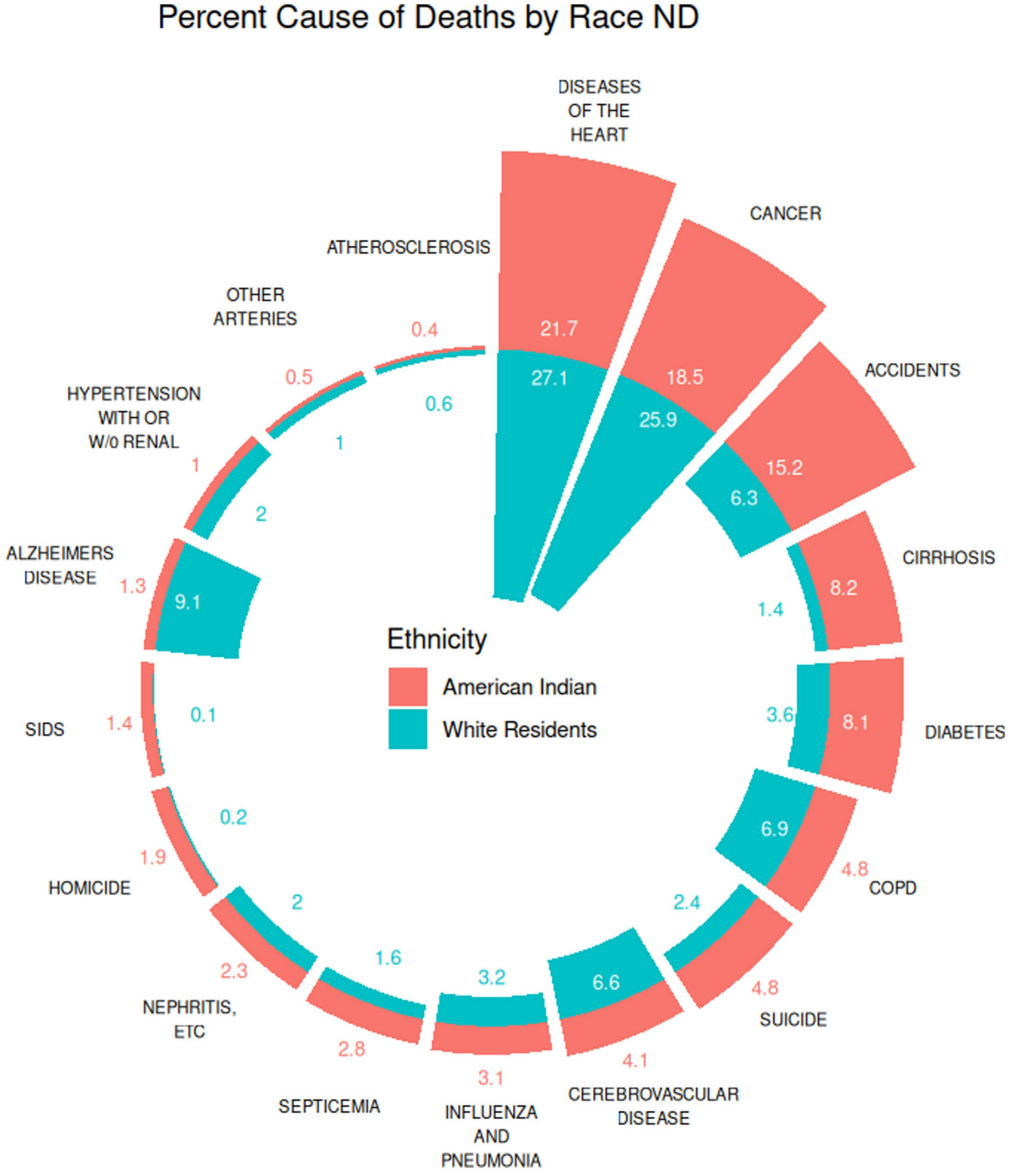
Percent cumulative death by cause, North Dakota 2010–2019.

A side-by-side percent comparison by cause of death for American Indian and White residents in North Dakota from 2010 to 2019 is demonstrated in Figure 3. The three leading causes of death for American Indians in North Dakota, accounting for approximately 55.4% of all deaths were diseases of the heart (21.7%), cancer (18.5%), and accidents (15.2%). The three leading causes of death for White residents in North Dakota during the same time period were diseases of the heart (27.1%), cancer (25.9%) and Alzheimer’s disease (9.1%), accounting for approximately 62.1% of all deaths. When looking further, from 2010 to 2019, all cause mortality rates (adjusted for age and stratified by gender) were higher in every age and sex stratum for American Indians compared to White residents in North Dakota. When looking at cause specific mortality rates during this same time period, American Indians in North Dakota exhibit significantly higher mortality rates for almost all age and sex stratum (Table 1). Some examples of these disparities are highlighted below. Death from accidents is significantly higher for both American Indian males and females, ages 1–4, 5–14, and 25-74 compared to their White counterparts. Similarly, mortality from homicide is significantly higher for American Indian males and females, age 5–44, compared to White males and females in this age range. There are only a few exceptions where the data, stratified by sex and age, demonstrate higher rates of cause specific mortality for White residents compared to American Indians.

**Table 1:** Age-specific death rates and rate ratios for common causes of death in ND.

Race	Ages 0–1	Ages 1–4	Ages 5–14	Ages 15–24	Ages 25–34	Ages 35–44	Ages 45–54	Ages 55–64	Ages 65–74	Ages 75–84	Age 85+
All accidents, V01-X59,Y85-Y86											
*Female*											
American Indian	–	26.00	51.00	479.00	500.00	497.00	404.00	267.00	601.00	1077.00	4023.00
White	62.00	22.00	22.00	58.00	97.00	116.00	101.00	108.00	122.00	428.00	1775.00
RR	–	**1.16**	**2.32**	**8.24**	**5.17**	**4.28**	**4.01**	**2.47**	**4.91**	**2.51**	**2.27**
*Male*											
American Indian	–	154.00	61.00	14.00	1112.00	989.00	1224.00	1143.00	1063.00	589.00	2062.00
White	96.00	33.00	31.00	196.00	255.00	264.00	265.00	296.00	371.00	834.00	2250.00
RR	–	**4.63**	**1.95**	**0.07**	**4.37**	**3.75**	**4.62**	**3.86**	**2.87**	**0.71**	0.92
Alzheimer’s disease, G300-G309											
*Female*											
American Indian	–	–	–	–	–	–	–	–	120.00	942.00	10345.00
White	–	–	–	–	–	–	5.00	13.00	147.00	1532.00	9707.00
RR	–	–	–	–	–	–	–	–	**0.82**	**0.62**	1.07
*Male*											
American Indian	–	–	–	–	–	–	–	–	304.00	393.00	2062.00
White	–	–	–	–	–	–	12.00	14.00	121.00	1259.00	7136.00
RR	–	–	–	–	–	–	–	–	**2.52**	**0.31**	**0.29**
Cancer, C00-C97											
*Female*											
American Indian	–	–	–	42.00	67.00	281.00	696.00	1897.00	4026.00	7402.00	13793.00
White	12.00	6.00	4.00	10.00	38.00	113.00	395.00	996.00	2445.00	4348.00	5830.00
RR	–	–	–	**4.28**	**1.77**	**2.48**	**1.76**	**1.90**	**1.65**	**1.70**	**2.37**
*Male*											
American Indian	92.00	26.00	24.00	27.00	49.00	112.00	724.00	2358.00	5543.00	11198.00	11340.00
White	12.00	9.00	6.00	17.00	28.00	116.00	374.00	1272.00	3229.00	7061.00	10798.00
RR	**7.68**	**2.83**	**3.75**	**1.64**	**1.75**	0.97	**1.94**	**1.85**	**1.72**	**1.59**	1.05
Cerebrovascular disease, I60-I69											
*Female*											
American Indian	189.00	–	–	–	50.00	65.00	112.00	296.00	541.00	3499.00	5747.00
White	25.00	–	–	1.00	6.00	3.00	42.00	59.00	301.00	1282.00	4896.00
RR	**7.54**	–	–	–	**8.48**	**22.60**	**2.67**	**5.00**	**1.80**	**2.73**	**1.17**
*Male*											
American Indian	–	26.00	–	14.00	16.00	45.00	275.00	214.00	835.00	1572.00	5155.00
White	–	–	–	1.00	4.00	14.00	61.00	127.00	365.00	1294.00	3974.00
RR	–	–	–	**13.92**	**4.08**	**3.18**	**4.52**	**1.69**	**2.29**	**1.21**	**1.30**
Cirrhosis, K70, K73-K74											
*Female*											
American Indian	–	–	–	–	150.00	627.00	628.00	534.00	661.00	269.00	575.00
White	–	–	–	–	7.00	33.00	83.00	70.00	45.00	104.00	68.00
RR	–	–	–	–	**21.20**	**18.99**	**7.59**	**7.63**	**14.85**	**2.58**	**8.46**
*Male*											
American Indian	–	–	–	14.00	278.00	652.00	899.00	1215.00	759.00	393.00	–
White	–	–	–	1.00	13.00	48.00	118.00	162.00	168.00	139.00	99.00
RR	–	–	–	**13.92**	**21.33**	**13.71**	**7.63**	**7.48**	**4.53**	**2.83**	–
COPD, J40-J47											
*Female*											
American Indian	–	–	–	–	–	–	67.00	326.00	1562.00	2961.00	6897.00
White	–	3.00	1.00	–	4.00	9.00	28.00	143.00	519.00	1546.00	2552.00
RR	–	–	–	–	–	–	**2.40**	**2.27**	**3.01**	**1.92**	**2.70**
*Male*											
American Indian	92.00	–	–	–	33.00	–	125.00	357.00	1367.00	3143.00	5155.00
White	–	–	–	2.00	3.00	4.00	32.00	177.00	680.00	2004.00	4278.00
RR	–	–	–	–	**10.87**	–	**3.95**	**2.02**	**2.01**	**1.57**	1.20
Diabetes, E10-E14											
*Female*											
American Indian	–	–	–	14.00	17.00	173.00	359.00	623.00	1623.00	3499.00	7471.00
White	–	–	–	–	4.00	16.00	41.00	71.00	219.00	579.00	1448.00
RR	–	–	–	–	**4.71**	**10.96**	**8.81**	**8.75**	**7.41**	**6.04**	**5.16**
*Male*											
American Indian	–	–	–	27.00	33.00	135.00	375.00	1215.00	2050.00	3536.00	5155.00
White	–	–	–	7.00	8.00	36.00	73.00	149.00	362.00	884.00	1831.00
RR	–	–	–	**3.98**	**4.08**	**3.75**	**5.14**	**8.16**	**5.67**	**4.00**	**2.81**
Diseases of the heart, I00-I09,I11,I13,I20-I51											
*Female*											
American Indian	94.00	–	–	–	83.00	303.00	673.00	1601.00	3305.00	6057.00	25287.00
White	75.00	3.00	1.00	4.00	18.00	65.00	140.00	336.00	1033.00	3352.00	16396.00
RR	**1.26**	–	–	–	**4.71**	**4.69**	**4.80**	**4.77**	**3.20**	**1.81**	**1.54**
*Male*											
American Indian	–	–	12.00	27.00	262.00	607.00	1499.00	3037.00	6378.00	9823.00	26804.00
White	36.00	–	–	8.00	37.00	141.00	406.00	977.00	2216.00	5927.00	19905.00
RR	–	–	–	**3.48**	**7.05**	**4.29**	**3.69**	**3.11**	**2.88**	**1.66**	**1.35**
Homicide, U01–U02, X85–Y09, Y87.1											
*Female*											
American Indian	–	26.00	13.00	42.00	67.00	43.00	–	–	60.00	–	–
White	12.00	–	3.00	3.00	4.00	10.00	8.00	7.00	–	5.00	4.00
RR	–	–	**4.64**	**12.84**	**18.84**	**4.30**	–	–	–	–	–
*Male*											
American Indian	92.00	–	24.00	109.00	278.00	90.00	75.00	143.00	–	–	–
White	24.00	6.00	8.00	13.00	16.00	21.00	11.00	11.00	6.00	4.00	–
RR	**3.84**	**–**	**3.12**	**8.56**	**17.33**	**4.37**	**6.85**	**12.58**	–	–	–
Influenza And Pneumonia, J10-J18											
*Female*											
American Indian	–	–	–	14.00	33.00	108.00	180.00	208.00	481.00	1750.00	4598.00
White	–	3.00	1.00	2.00	4.00	13.00	22.00	57.00	95.00	491.00	1992.00
RR	–	–	–	**6.42**	**9.42**	**8.37**	**8.29**	**3.65**	**5.08**	**3.56**	**2.31**
*Male*											
American Indian	184.00	26.00	–	–	49.00	67.00	150.00	179.00	304.00	1768.00	–
White	12.00	–	1.00	3.00	4.00	6.00	23.00	66.00	181.00	596.00	2817.00
RR	**15.37**	–	**–**	**–**	**12.23**	**10.49**	**6.49**	**2.71**	**1.68**	**2.97**	–
Septicemia A40-A41											
*Female*											
American Indian	94.00	–	–	28.00	33.00	65.00	180.00	296.00	361.00	538.00	1149.00
White	–	–	–	3.00	6.00	9.00	20.00	49.00	134.00	255.00	590.00
RR	–	–	–	**8.56**	**5.65**	**7.53**	**8.81**	**6.10**	**2.70**	**2.11**	**1.95**
*Male*											
American Indian	–	–	–	–	114.00	90.00	200.00	214.00	607.00	589.00	2062.00
White	–	–	–	–	3.00	13.00	24.00	51.00	177.00	314.00	928.00
RR	–	–	–	–	**38.06**	**6.99**	**8.23**	**4.19**	**3.43**	**1.88**	**2.22**
SIDS R95											
*Female*											
American Indian	1320.00	–	–	–	–	–	–	–	–	–	–
White	225.00	–	–	–	–	–	–	–	–	–	–
RR	**5.87**	–	–	–	–	–	–	–	–	–	–
*Male*											
American Indian	2208.00	–	–	–	–	–	–	–	–	–	–
White	275.00	–	–	–	–	–	–	–	–	–	–
RR	**8.02**	–	–	–	–	–	–	–	–	–	–
Suicide, X60-X84, X87.0											
*Female*											
American Indian	–	–	63.00	127.00	200.00	130.00	90.00	30.00	–	–	–
White	–	–	4.00	31.00	48.00	52.00	59.00	43.00	17.00	14.00	4.00
RR	–	–	**15.47**	**4.13**	**4.14**	**2.51**	**1.53**	**0.69**	–	–	–
*Male*											
American Indian	–	–	12.00	519.00	392.00	292.00	325.00	214.00	76.00	–	–
White	–	–	6.00	156.00	183.00	175.00	214.00	167.00	100.00	153.00	140.00
RR	–	–	**1.87**	**3.33**	**2.14**	**1.67**	**1.52**	**1.28**	**0.76**	–	–
All Causes, A00-Y99											
*Female*											
American Indian	5184.00	179.00	165.00	859.00	1615.00	3006.00	4421.00	7857.00	16346.00	35666.00	97126.00
White	2362.00	79.00	49.00	136.00	300.00	583.00	1271.00	2502.00	6484.00	18107.00	60962.00
RR	**2.19**	**2.27**	**3.35**	**6.32**	**5.38**	**5.16**	**3.48**	**3.14**	**2.52**	**1.97**	**1.59**
*Male*											
American Indian	6624.00	435.00	182.00	902.00	3172.00	3844.00	7394.00	12612.00	23538.00	39489.00	84536.00
White	2718.00	103.00	70.00	445.00	656.00	1013.00	2018.00	4452.00	9835.00	26171.00	72557.00
RR	**2.44**	**4.24**	**2.60**	**2.03**	**4.84**	**3.79**	**3.66**	**2.83**	**2.39**	**1.51**	1.17

RR values in bold are significant *p* < 0.05.

ASDR is scaled as deaths per 100,000 residents.

ASDR was only computed for raw counts greater than 10.

ND represents North Dakota.

Entries listed as—represent non-computed values.

ASDR, Age Specific Death Rate.

## 4 Discussion

### 4.1 Disparities in mortality causes among American Indians in North Dakota

Understanding the differences in risks between American Indians and their White counterparts is critical, as American Indians in North Dakota exhibit higher RRs for all common causes of death (adjusted for age and stratified by gender) from 2010 to 2019 (see Table 1), a few of which are highlighted below.

*Influenza and Pneumonia*: American Indians in North Dakota were significantly impacted by influenza and pneumonia from 2010 to 2019 with American Indians experiencing RRs up to 15 times higher than White residents. Among females, the highest RR is observed in the 25–34 age group, where American Indian females have a RR 9.42 times higher than White females. For males, the highest RR is seen in the 0–1 age group, where American Indian males have a rate 15.37 times higher than White males, highlighting a significant early-life risk. Compared to the general U.S. population, American Indians and Alaska Natives are 1.8 times more likely to die from influenza and pneumonia (6). It is thought that social and economic factors, such as insufficient funding for the Indian health care system, result in inadequate access to health care (6). The data also highlights the need for targeted health interventions to address these disparities and reduce the rates of influenza and pneumonia among American Indians in North Dakota.
*Septicemia*: American Indians in North Dakota were significantly impacted by septicemia from 2010 to 2019 with American Indians experiencing RRs up to 38 times higher than White residents. American Indian females ages 45–54 have a RR 8.81 times higher than White females. For males, the highest RR is seen in the 25–34 age group, where American Indian males have a rate 38.06 times higher than White males, highlighting an extremely high disparity. A study by Prest et al. published in 2022 evaluated mortality rates from sepsis by race from 2004 to 2018 and found that American Indians had the highest mortality rates per 100,000 people of all racial groups for abdominal sepsis 66.9 (63.7–70.0) and the second highest mortality rates per 100,000 people for pulmonary sepsis 163.2 (158.2–168.1) and urogenital sepsis 63.6 (60.4–66.9) (7). Septicemia is a critical health concern for American Indians, who already face significantly elevated mortality rates from pneumonia, highlighting the urgent need for targeted interventions to address these interconnected health risks.
*Suicide*: American Indians in North Dakota were significantly impacted by suicide from 2010 to 2019 with American Indians experiencing RRs up to 15 times higher than White residents. Among females the highest RR is observed in the 5–14 age group, where American Indian females have a rate 15.47 times higher than White females. For males, the highest risk is seen in the 15–24 age group, where American Indian males have a RR 3.33 times higher compared to White males. A comparison of suicide rates from 2015 to 2020 among American Indians and Alaska Natives and non-American Indian/Alaska Native decedents across 49 states, Puerto Rico, and the District of Columbia found that suicide rates for non-Hispanic American Indian and Alaska Native individuals rose nearly 20%, from 20.0 per 100,000 in 2015 to 23.9 in 2020, compared to a less than 1% increase for the overall U.S. population, which went from 13.3 to 13.5 (8). The authors found that American Indian and Alaska Native suicide decedents had significantly higher adjusted odds across a range of circumstances preceding the suicide which included the following: suicide event or history (disclosed suicidal intent, history of suicidal thoughts or plan), relationship problem (family, intimate, non-intimate, interpersonal violence within previous month, or an argument or conflict preceded death), loss (suicide of friend or family member), other life stressors (victim in custody; jail, prison, or a detention facility; criminal or legal problem), crisis within previous 2 weeks or anticipated in upcoming 2 weeks (related to the following problems: alcohol, intimate partner, criminal legal, recent suicide of friend or family), and substance use compared to non-American Indian or Alaska Native persons (8). The authors also found that nearly 75% of American Indian and Alaska Native suicide decedents were 44 years of age or younger compared to less than half (45.6%) of non-American Indian/Alaska Native decedents (8). Culturally relevant comprehensive public health approaches are necessary to address these disparities and reduce the rates of suicide among American Indians in North Dakota and ensure that American Indian communities receive the support and resources needed for better health outcomes. Furthermore, providers and program administrators should work to ensure that they are delivering culturally responsive services. The Substance Abuse and Mental Health Services Administration (SAMHSA) has created a Treatment Improvement Protocol (TIP) 61 titled Behavioral Health Services for American Indians and Alaska Natives For Behavioral Health Service Providers, Administrators, and Supervisors which is designed to serve as a primer for working with American Indians and Alaska Natives to help providers and program administrators provide culturally responsive, engaging, holistic, trauma-informed behavioral health services (9). TIP 61 provides a multitude of key messages for providers and program administrators to focus on including (1) the importance of historical trauma, (2) acceptance of a holistic view of behavioral health, (3) role of culture and identity, (4) recognition of sovereignty, (5) significance of community, and (6) the value of cultural awareness (9).
*Homicide:* American Indians in North Dakota were significantly impacted by homicide from 2010 to 2019 with American Indians experiencing RRs up to 18 times higher than White residents. Among American Indian females, the highest risk is found at ages 25–34 with a RR of 18.84 compared to White females. Among American Indian males, the highest risk is seen in ages 25–34 with a RR of 17.33. A Nationwide study among 34 states and the District of Columbia evaluated homicide patterns for American Indians and Alaska Natives from 2003 to 2018 noting a multitude of precipitating circumstances that impact homicide including mental health/substance use, interpersonal, life stressor, crime/criminal activity or homicidal events (10). The authors found that the median age of the American Indian and Alaska Native victims was 32 years and also demonstrated that “homicides of AI/AN males, particularly among youths and young adults, contribute to many AI/AN homicides” (10). A multitude of prevention strategies have been identified to address violence in American Indian communities. Prevention efforts should consist of interventions designed to lesson harms and prevent future risk, connect youth to caring adults and activities, teach safe and healthy relationship skills, teach coping and problem-solving skills, promote family environments that support healthy development, promote connectedness, and create protective community environments (11). Tribal communities have developed programs that also uphold Tribal teachings and traditions (11).

Overall, for American Indians in North Dakota, death rates were significantly higher, from 2010 to 2019, starting at birth and resulting in a life expectancy gap of 22.5 years. American Indians face significant disparities for all common causes of death in North Dakota throughout the lifespan. There are only a few exceptions where the data, stratified by sex and age, demonstrate higher rates of cause specific mortality for White residents compared to American Indians.

### 4.2 American Indians across the United States

American Indian populations throughout the U.S. are diverse and strong with unique cultures, languages, and traditions. There are currently 574 federally recognized Tribes within the U.S. (12). With an estimated 9.7 million people identifying as American Indian or Alaska Native, alone or in combination (more than one race), American Indians and Alaska Natives represent about 3% of the total U.S. population (13). The premature mortality of American Indians is alarming and has increased steadily in the U.S. while declining among other minoritized populations (14). Although this study focuses on the decade prior to the pandemic, recent reports from the Center for Disease Control National Center for Health Statistics shows American Indian and Alaska Native populations had the biggest drop in life expectancy in 2021 of 1.9 years, with a new top-ten mortality outcome due to COVID-19. American Indians had a life expectancy at birth of 65.2 years in 2021, equal to the life expectancy of the total U.S. population in 1944 (15). The life expectancy for American Indian and Alaska Native populations has declined 6.6 years from 2019 to 2021 (16). Previous studies indicate that premature mortality among American Indian and Alaska Native populations is expected to continue to increase through 2030 (17). During 2019–2030, all-cause deaths are projected to increase among American Indians and Alaska Natives (14).

### 4.3 American Indians and social determinants of health in North Dakota

Social Determinants of Health (SDOH) factors such as economics, education, health care, environment, and community significantly impact the overall health and well-being of an individual (18). According to Healthy People 2030, SDOH are “the conditions in the environments where people are born, live, learn, work, play, worship, and age that affect a wide range of health, functioning, and quality-of-life outcomes and risks” (18). Across the nation, it is estimated that 87% of the American Indian/Alaska Native population live in urban areas (19). In North Dakota, less than 40% of American Indians live in urban areas with more than 60% residing on reservations (19). SDOH impacting the American Indian population include, but are not limited to, lack of education, a lack of health insurance coverage and poverty (20). American Indians in North Dakota are less likely to have health insurance coverage (72.0%) compared to White residents (94.0%) (21). From 2015 to 2019, North Dakota experienced a poverty rate of (10.6%), ranking it the 10^th^ lowest poverty rate in the country (22). However, overall American Indians in the state experience a notably higher poverty rate (31.0%) (23). The counties in North Dakota with the highest rates of poverty are Sioux County (42.8%), Rolette County (27.2%), and Benson County (26.3%) (22). These counties include all or part of the Standing Rock Indian Reservation, Turtle Mountain Indian Reservation, and Spirit Lake Indian Reservation, respectively. During the period of 2018–2022, the overall “all families” poverty rates for Tribes in North Dakota were as follows: Spirit Lake Nation (33.1%), Standing Rock Sioux Tribe (32.4%), Turtle Mountain Band of Chippewa Indians (21.2%), and Fort Berthold Reservation (14.3%) (24). Research has found that low socioeconomic status is associated with increased mortality, even after accounting for a multitude of individual level behavioral factors including smoking, alcohol consumption, and physical activity (25).

### 4.4 Addressing overall American Indian health in North Dakota

Overall health of American Indians in North Dakota should be addressed more broadly, rather than with a disease-specific approach. This study further characterizes the severity of the disparities that exist among American Indians in North Dakota and is a call to action for North Dakota communities, health care providers, institutions, including public health and academic, as well as policymakers.

Data from the Indian Health Service (IHS) suggest that lower life expectancy (5.5 years less than other races) for American Indians is longstanding (6). Further, IHS data reveal specific disparities for chronic diseases such as heart disease, cancer, and diabetes, as well as unintentional injuries as the overall highest causes of death for American Indians, in the U.S. (6). Recognizing these disparities within American Indian communities should be seen as an opportunity for improvement to the health system in North Dakota.

A multitude of factors impact the overall health and well-being of American Indians in North Dakota. Rural and remote areas are often more isolated, lacking healthcare infrastructure and capacity, and American Indian or Alaska Native individuals will sometimes delay seeking care in part because they do not trust organizations (9). Other factors that influence American Indian people from seeking available care include lack of transportation, perceived provider effectiveness and geographic location (9). Despite demonstrating resilience, American Indians in North Dakota still face persistent health disparities and systemic violence, with ongoing social, economic, and environmental factors exacerbating existing health inequities. Social and economic disparities that have historically impacted Tribal populations include, but are not limited to, colonization, forced migration, land loss, and cultural devastation (26).

Understanding and addressing these social determinants is crucial for developing effective strategies to improve the health and well-being of American Indian and Alaska Native populations, particularly American Indians living in rural regions of North Dakota. Given these disparities, policy makers should ensure that individuals suffering at an early age due to poor health should have an approach streamlined for them so that they can access much needed support and financial resources.

### 4.5 Limitations

When reviewing mortality for American Indians, it is important to recognize that death certificates are relied upon for data. Misclassification of race and ethnicity on U.S. death certificates has been a challenge affecting public health data for American Indians (27). Census misclassification issues impacting population estimates also present problems with regard to mortality statistics (28). When data are adjusted to account for this misclassification, American Indian mortality rates are even higher than those demonstrated here (29).

## 5 Conclusion

Disparities in mortality between American Indian and White residents in North Dakota are large and egregious. To best address the health and wellbeing of American Indians in North Dakota, multisectoral efforts focused on prevention, improved policies, and cultural humility and safety in the health systems are needed. The health of American Indian communities depends on the collaborative efforts of policymakers, healthcare professionals, health care systems and tribal governing bodies. Solutions should center American Indian voices, cultures and spaces, and include Tribes, Tribal organizations, Tribal health, Indian Health Service and Urban Indian Health Centers. Additionally, standards for death certificates in the US could benefit populations by accurately reflecting race and rates of illness and death.

## Data Availability

Publicly available datasets were analyzed in this study. Authors requested data from the North Dakota Data Center and North Dakota State Health Department.
